# Mucosa associated lymphoid tissue lymphoma presenting within a solitary anti-mesenteric dilated segment of ileum: a case report

**DOI:** 10.1186/1752-1947-3-6

**Published:** 2009-01-06

**Authors:** Rowland Storey, Marcel Gatt, Ian Bradford

**Affiliations:** 1 Department of General Surgery, York Hospital, Wigginton Road, York, YO31 8HE, UK

## Abstract

**Introduction:**

Mucosa associated lymphoid tissue (MALT) lymphoma is the third most common non-Hodgkin's lymphoma subtype. Clinical presentation is often insidious as a low-grade lesion and disease tends to remain localised for a long period of time. Ileal involvement is rare and presentation within an area of focal anti-mesenteric ileal wall dilation simulating a large diverticulum has not been reported.

**Case presentation:**

A 59-year-old man of Caucasian origin presented to a general surgical outpatients clinic with an 18-month history of intermittent upper abdominal pain following meals. Following normal gastroscopy and abdominal ultrasound, a focally dilated segment of ileum was seen on computed tomography and further clarified by barium investigation. Histology of this segment demonstrated MALT lymphoma of the small bowel.

**Conclusion:**

A solitary focally dilated segment of ileal wall may be neoplastic in nature and surgical resection needs to be considered.

## Introduction

Mucosa associated lymphoid tissue (MALT) lymphoma otherwise known as extra-nodal marginal zone lymphoma was first described by Isaacson and Wright in 1983. The term MALT lymphoma was originally used to describe lymphoid tissue seen to histologically closely resemble that of the terminal ileum [1]. MALT lymphoma is the third most common non-Hodgkin's lymphoma subtype and often presents insidiously as a low-grade lesion which tends to remain localised for a long period of time [2]. The gastrointestinal tract, but in particular the stomach, is by far the most common extra-nodal site. MALT lymphoma of the ileum, however, is rare. Little is known with certainty about the pathogenesis of the disease but overall 5-year survival for MALT lymphoma is reported as 81% [3].

## Case presentation

**Figure 1:**
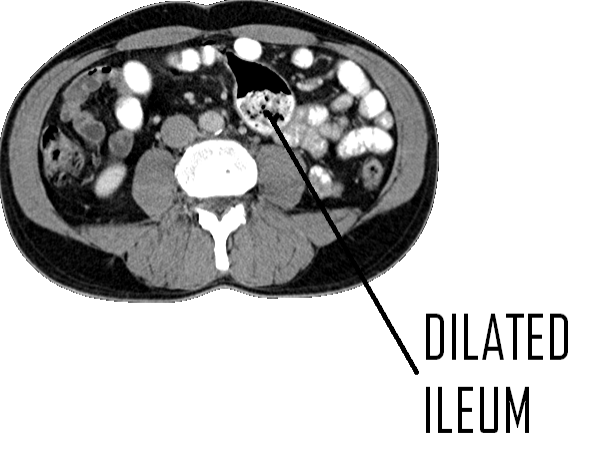
**Computed tomography displaying a dilated segment of ileum (indicated by the arrow)**.

**Figure 2:**
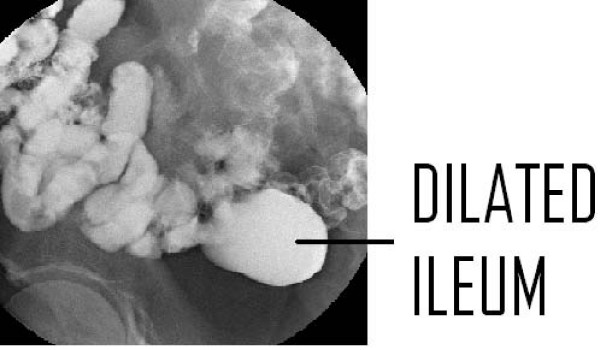
**Barium meal and follow through demonstrating a dilated ileal segment (indicated by the arrow)**.

This report describes the case of a 59-year-old Caucasian man presenting to a general surgical outpatients clinic with an 18-month history of intermittent upper abdominal pain following meals. Clinical examination, blood tests and an ultrasound examination were all unremarkable and no justification for symptoms was found at upper gastrointestinal endoscopy. A computed tomography (CT) scan of the abdomen following an acute admission with abdominal pain and vomiting demonstrated a 4.6 cm dilated segment of ileum containing both contrast and debris (Figure 1). This was further clarified and shown to be solitary by barium meal and follow through (Figure 2). Differential diagnoses included Meckel's diverticulum, non-Meckel's diverticulum and ileal duplication. A decision was made to proceed to laparoscopy with a view to segmental small bowel resection.

**Figure 3:**
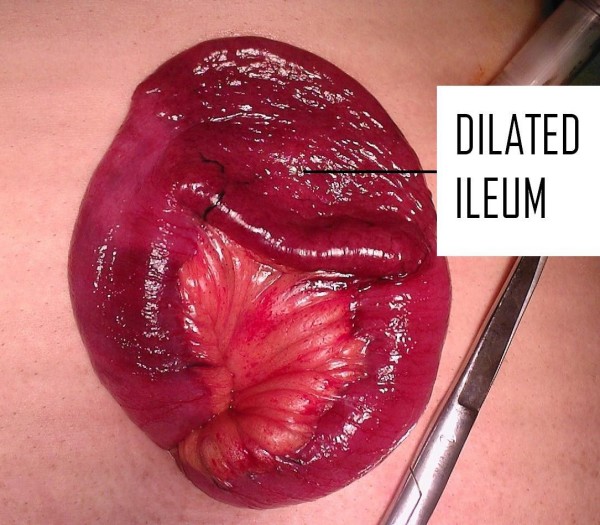
**Operative view of the specimen delivered through a laparoscopic port**. The abnormal segment of ileum (arrow) can be seen to be dilated in comparison to adjacent normal ileum.

At laparoscopy, a focally dilated anti-mesenteric segment of ileum was found 140 cm from the ileocaecal valve and displaying an abnormal serosa (Figure 3). A segmental resection with side-to-side anastomosis was performed through an enlarged umbilical port site incision. No lymphadenopathy within the mesentery was apparent. Following haematoxylin and eosin staining, extensive infiltration by small monomorphic lymphoid cells was seen on histological examination. No ectopic mucosa was identified. Immunohistochemical analysis revealed atypical cells positive for CD20 and CD79a, and negative for CD3, CD5, CD10 and cyclin D1. A diagnosis of MALT lymphoma was made. Subsequent bone marrow examination and staging CT showed no evidence of disseminated disease staging the lymphoma as 1E using the revised European-American Lymphoma clinic staging system [4].

## Discussion

A thorough literature review has failed to identify a similar case of MALT lymphoma presenting within an area of focal anti-mesenteric dilation simulating a large diverticulum. Other differentials include MALT within a non-Meckel's diverticulum, ileal duplication or Meckel's diverticulum. Small bowel diverticula (non-Meckel's) most commonly arise in the duodenum and emerge on the mesenteric border where mesenteric vessels penetrate the bowel wall. Intestinal duplication is a rare condition which tends to present in early life and is closely associated with the mesenteric border. Isolated adult cases of ileal duplication, some with malignant change, have however been reported [5]. Intestinal duplication and non-Meckel's ileal diverticulum are unlikely differential diagnoses. A Meckel's diverticulum is however found on the mesenteric border and is an important differential diagnosis. Classically, a Meckel's diverticulum is seen 40 to 60 cm proximal to the ileo-caecal valve. The case we describe was 140 cm from the ileo-caecal valve. In addition, no ectopic mucosa was found within the specimen although only 43% of symptomatic adults have ectopic mucosa within a Meckel's diverticulum [6]. Consequently, we cannot either confirm or exclude the potential for MALT lymphoma as a complication of a Meckel's diverticulum based on histology. The location of the pathology in question is however in an atypical position for a Meckel's diverticulum. To our knowledge, MALT lymphoma of a Meckel's diverticulum has also not been reported.

MALT lymphoma of the GI tract often presents with non-specific symptoms. Additionally, traditional investigative techniques often miss the early stages of the diseases process making timely clinical diagnosis challenging. In patients with unexplained gastrointestinal symptoms, video capsule endoscopy may need to be considered as it is the diagnostic test with the highest yield for small bowel malignancies [7].

## Conclusion

A solitary focally dilated segment of ileal wall may be neoplastic in nature and surgical resection needs to be considered.

## Abbreviations

CT: computed tomography; MALT: mucosa associated lymphoid tissue

## Consent

Written informed consent was obtained from the patient for publication of this case report and any accompanying images. A copy of the written consent is available for review by the Editor-in-Chief of this journal.

## Competing interests

The authors declare that they have no competing interests.

## Authors' contributions

RS, MG and IB were all involved in the drafting and re-editing of the manuscript. The final manuscript was read and approved by all three authors.
